# Effects of Various Decellularization Methods for the Development of Decellularized Extracellular Matrix from Tilapia (*Oreochromis niloticus*) Viscera

**DOI:** 10.1155/2024/6148496

**Published:** 2024-09-18

**Authors:** Jemwel Aron, Ronald Bual, Johnel Alimasag, Fernan Arellano, Lean Baclayon, Zesreal Cain Bantilan, Gladine Lumancas, Michael John Nisperos, Marionilo Labares, Kit Dominick Don Valle, Hernando Bacosa

**Affiliations:** ^1^Environmental Science Graduate Program-Department of Biological Sciences, MSU-Iligan Institute of Technology, Iligan City 9200, Philippines; ^2^Chemical Engineering Department, University of San Agustin, Iloilo City 5000, Philippines; ^3^Center for Sustainable Polymers, MSU-Iligan Institute of Technology, Iligan City 9200, Philippines; ^4^Department of Chemical Engineering and Technology, College of Engineering, MSU-Iligan Institute of Technology, Iligan City 9200, Philippines

## Abstract

Tilapia, a widely farmed aquaculture fish, produces substantial waste, including viscera that contain extracellular matrix (ECM) utilized as a biomaterial for tissue regeneration applications. Extracting ECM from viscera requires a specific decellularization method, as no standardized protocol exists. This study performed three decellularization methods: sonication, orbital shaking at room temperature, and agitation at 4°C, using SDS and TX100 at concentrations of 0.1% and 0.3%. The effectiveness of each method was assessed through H&E staining, dsDNA quantification, and SEM imaging to verify cellular content removal and ECM structure preservation. Additional analyses, including ATR-FTIR, SDS-PAGE, protein quantification, HPLC, and detergent residue tests, were performed to examine functional groups, collagen composition, protein content, amino acid profiles, and detergent residues in the decellularized samples. The results of H&E staining showed a significant reduction in cellular components in all samples, which was confirmed through DNA quantification. Sonication with 0.3% SDS achieved the highest DNA removal rate (96.5 ± 1.1%), while SEM images revealed that agitation at 4°C with 0.3% TX100 better preserved ECM structure. Collagen was present in all samples, as confirmed by ATR-FTIR analysis, which revealed pronounced spectral peaks in the amide I, II, III, A, and B regions. Samples treated with agitation at 4°C using 0.1% SDS exhibited the highest protein content (875 ± 15 *µ*g/mg), whereas those treated with TX100 had lower detergent residue. Overall, the decellularization methods effectively reduced DNA content while preserving ECM structure and components, highlighting the potential of tilapia viscera as bioscaffolds and offering insights into utilizing fish waste for high-value products.

## 1. Introduction

Tilapia (*Oreochromis niloticus*) is a widely farmed fish species in aquaculture. It is valued for its high protein content and disease resistance [1, 2]. However, the growing importance of tilapia in the fish processing industry has resulted in the production of substantial waste, raising environmental concerns, particularly regarding water pollution. By-products from tilapia processing include fins, gills, backbones, heads, skin, and viscera [3–5], which are rich sources of proteins, minerals, enzymes, collagen, and other bioactive molecules [6, 7]. Among these, collagen has garnered significant attention due to its potential applications in regenerative medicine [8]. Recent studies, such as the one by Cao et al. [9], have highlighted the development of innovative products like composite skin patches using acellular fish skin from tilapia combined with chitosan for wound healing. Another study by Liu and Sun [10] introduces hydrolyzed tilapia fish collagen (HFC) as a promising bioactive ingredient for periodontal tissue regeneration. Stone et al. [11] studied the effects of acellular fish skin versus acellular cowhide on burn repair and concluded that acellular fish skin was more effective for this purpose. These advancements have sparked further research into the potential biomedical applications of other fish byproducts.

Among the components of fish waste, the viscera, which comprise 12% to 18% of a fish's total weight, are often discarded, causing water pollution, aesthetic degradation, and other environmental issues [12]. The viscera organ, composed of the gut, liver, spleen, stomach, heart, and pancreas, contains functional compounds such as proteins, lipids, amino acids, vitamins, minerals, and omega-3 fatty acids [13, 14]. The abundant quantity of viscera has garnered significant interest in developing products such as fish viscera oil, digestive enzymes, protein, and its derivatives [15]. Despite some of these products being undervalued and less attractive in the market tilapia viscera, like other connective tissues, contain a complex network of proteins known as the extracellular matrix (ECM) [12, 16]. This ECM provides structural support and serves as a valuable biomaterial and bioscaffold used in tissue regeneration and biomedical applications [17–19].

Various laboratory techniques have been utilized to extract valuable compounds from fish viscera. While most processes primarily target oil and protein extraction [15], no established methods are specifically designed to extract the extracellular matrix (ECM) and its associated components for biomaterial advancement applications. Driven by this potential, one of the techniques being considered for extracting the ECM from tissues is decellularization. This noteworthy technique removes immunogenic cellular components while preserving the underlying ECM structure [20]. Decellularization protocols vary and are typically tailored based on tissue-specific factors like density and geometry [4, 18, 21, 22]. For thinner tissues, the extent of cellular removal depends on the intensity of agitation or exposure to relatively mild detergent concentrations [23]. Although physical treatments can aid in the removal of cellular material, they are not sufficient to achieve complete decellularization on their own. For optimal results, physical methods are typically combined with chemical, biological, and enzymatic agents [24]. The combination of physical treatments along with other agents, such as freeze-thawing with Triton X-100 and Sodium Dodecyl Sulfate (SDS), has been shown to effectively extract extracellular matrix (ECM) with minimal alteration in its composition, structure, and mechanical properties. Azhim et al. [25] utilized sonication combined with SDS to decellularize porcine aorta, while Kamalvand et al. [26] reported that TX100 yielded the best ECM in terms of structure, toxicity, cell adhesion, and mechanical and physical properties. However, despite these advancements, challenges persist in developing effective decellularization techniques, requiring further consideration.

Numerous studies have explored marine-derived extracellular matrix (ECM) decellularization from sources like tilapia skin [27, 28] and scales [29, 30]. However, there has been an absence of research investigating the potential use of fish viscera as a marine-derived ECM. Additionally, only a limited number of studies have assessed the effectiveness of combining physical methods and chemical detergents. To fully harness the potential of viscera waste, this study aimed to produce a decellularized ECM from tilapia viscera waste and determine, through three decellularization methods—sonication, orbital shaking at room temperature, and agitation at 4°C—combined with decellularizing agents (SDS and TX100) at concentrations of 0.1% and 0.3%, the appropriate protocols for effective decellularization of the viscera. To the best of our knowledge, this study marks the first attempt at utilizing tilapia viscera waste to assess its suitability as a valuable biomaterial for various applications, including tissue engineering, regenerative medicine, and pharmaceutical development. This study provides essential foundational data for successful tilapia viscera waste decellularization and the development of high-value products.

## 2. Materials and Methods

### 2.1. Sample Collection and Preparation

Fresh tilapia fish acquired from a local fish landing were washed and rinsed under cold running water to remove dirt sediments. Subsequently, the stomach of the tilapia was carefully lacerated to collect the viscera tissue. The collected viscera tissues were washed with cold running water to eliminate adhering contents, cut into sections, and immersed in phosphate-buffered saline (1X PBS, pH 7.4) solution for 1 hour. The prepared viscera were then stored in a freezer until further processing.

### 2.2. Decellularization of Viscera Tissue

Three decellularization methods were employed in this study: (1) sonication, (2) orbital shaking, and (3) agitation. These methods were based on and slightly modified from the protocols outlined by Azhim et al. [25] and Oliveira et al. [4]. Two different decellularizing agents, SDS (Loba Chemie, Mumbai, India) and TX100 (Loba Chemie, Mumbai, India), were used at concentrations of 0.1% and 0.3% in combination with each decellularization method. Hence, twelve physicochemical protocols (*n* = 3) were conducted to produce viscera-derived decellularized extracellular matrix (dECM), as detailed in Table 1.

For each protocol, 10 g of viscera sample was weighed and immersed in a beaker containing 100 mL of the decellularizing agent at a ratio of 1 : 10 (w/v). In the first method, the samples were subjected to sonication using a 40 kHz water bath sonicator (YTK Yason, Shenzhen, China) for 10 hours at room temperature (RT). To ensure consistent temperature control and prevent overheating, a pump and two thermometers were used to monitor and manage any temperature fluctuations during the sonication process. In the second method, decellularization was achieved using an orbital shaker (Jintan Medical Instrument Factory, China). The samples were agitated on the shaker for 24 hours at RT. The last method involved decellularization by agitation using a magnetic stirrer set at 300 rpm for 24 hours at 4°C. The samples were then washed thrice with deionized water for 15 minutes each wash, followed by an additional 24-hour wash. Subsequently, the decellularized samples were stored at −80°C Ultra-Low Temperature (ULT) for 24 hours before being freeze-dried using a freeze dryer (Gyrozen, Gimpo, South Korea) for another 24 hours. Finally, the freeze-dried samples or the decellularized extracellular matrix (dECM) were stored at 4°C for characterization.

### 2.3. Histological Staining Analysis

The dECM was subjected to histological analysis using the Hematoxylin and Eosin (H&E) staining method, as described by Bual et al. [31] with slight modifications. Tilapia viscera samples (around 5 × 5 mm^2^) weighing 0.70 g were fixed in 7 ml of 10% neutral buffered formalin solution at a ratio of 1 : 10 (w/v) for 72 hours, then rinsed with running distilled water for 1 hour. The fixed samples were dehydrated using a series of alcohol immersions with increasing concentration (70%, 95%, and 100%) for 1 hour at each concentration. Following dehydration, the samples were immersed three times in xylene for 30 minutes each before being embedded in paraffin wax blocks. The paraffin blocks were cut into 4 µm-thick ribbons using a microtome (Slee CUT 4062, Nieder-Olm, Germany). These ribbons were mounted on glass slides and rehydrated by alcohol immersion of decreasing concentrations—100%, 95%, and 70% ethanol—each for 3 minutes. The sections were stained with Hematoxylin and Eosin (Biognost®, Zagreb, Croatia) to differentiate basic cell components and acidic structures. Finally, the slides were dehydrated, cleared, mounted with coverslips, and examined under a phase-contrast microscope (Olympus CX43, Tokyo, Japan) at 100x magnification.

### 2.4. Scanning Electron Microscopy (SEM)

The dECM samples were mounted onto small pieces of adhesive carbon tape fixed on a brass stub. The samples were then subjected to gold coating using a sputtering unit (JEOL Smart Coater, Tokyo, Japan) for 1 minute. Subsequently, the gold-coated sample was placed in the chamber of the SEM (JSM-IT200, Tokyo, Japan), and the images were recorded.

### 2.5. Double-Stranded DNA (dsDNA) Quantification

To quantify the presence of residual DNA in the samples, the dECM was comminuted and weighed to approximately 25 mg each (*n* = 3). DNA extraction was performed on the samples using the DNeasy Blood and Tissue Kit (Qiagen®, Valencia, CA, USA). Then, the extracted DNA was quantified using the QubitTM 1X dsDNA HS Assay Kit (Thermo Fisher Scientific, Waltham, Massachusetts, USA), following the instructions and procedures provided by the manufacturer.

### 2.6. Total Protein Quantification

The protein content of the samples was determined by solubilizing 50 mg of lyophilized dECM sample (*n* = 3) in 5 mL of 0.1 M acetic acid containing 10 mg of pepsin (Merk, St. Louis, MO, USA) at 4°C for 48 hours. The protein content was determined using the Qubit Protein Assay Kit (Thermo Fisher Scientific, Massachusetts, USA) and quantified using a Qubit Fluorometer (Thermo Fisher Scientific, Massachusetts, USA).

### 2.7. Attenuated Total Reflectance-Fourier Transform Infrared (ATR-FTIR) Spectroscopy

ATR-FTIR spectroscopy was conducted to examine the presence of functional groups in the dECM and raw samples. The freeze-dried dECM samples were positioned on a plate and analyzed using a QATR-10 single reflection FTIR instrument (Shimadzu, Kyoto, Japan). The instrument was set up to perform 128 scans across a spectral range of 400 to 4000 cm^−1^ with a resolution of 4 cm^−1^.

Amide I peak fitting was conducted to estimate changes in the distribution of secondary structures of proteins in the extracellular matrix. Baseline corrections were performed using a straight-line method, with anchor points identified by the zero-crossings of the second derivative method. Peak fitting was performed on the FFT-smoothed Amide I spectra (3-point smoothing) using the Voigt function, a convolution of Gaussian and Lorentzian functions [32]. Baseline and peak center parameters were selected to initialize the fitting process. Iterations were stopped once the best fit was achieved, indicated by a reduced chi-square value of less than 1 × 10^−6^. The secondary structural content was estimated by dividing the areas under the peaks assigned to specific secondary structures by the total area under the Amide I band, with results reported as percentages.

### 2.8. Sodium Dodecyl Sulfate-Polyacrylamide Gel Electrophoresis (SDS-PAGE)

SDS-PAGE was performed according to the method of Laemmli [33] with minor modifications. Each dECM sample (50 mg each, *n* = 3) was solubilized in 5 mL of 0.05 M acetic acid containing 10 mg of pepsin (Merk, St Louis, MO, USA) for 48 hours at 4°C. Subsequently, the solubilized samples were subjected to gel electrophoresis using a stacking gel of 5% and a resolving gel of 7.5%. These gels were immersed in a Tris-HCl buffer at pH 6.8, comprising 0.6 M Tris-HCl, 25% (v/v) glycerine (Promega, Madison, Wisconsin), 2% (w/v) SDS, 5% (v/v) *β*-mercaptoethanol, and 0.1% (w/v) Bromophenol blue (Sigma-Aldrich, Missouri, USA). Subsequently, 10 *µ*L of the sample solution was loaded into each well. Electrophoresis was performed using a mini vertical protein electrophoresis system (omniPAGE CVS10DSYS-CU, Cleaver Scientific, United Kingdom) with a set voltage of 200 V and 20 mA for 2 hours. After electrophoresis, the gels were stained by soaking in 20 mL of 0.1% (v/v) Coomassie Brilliant Blue solution (Abcam, Massachusetts, USA) for 2 hours using an orbital shaker until clear bands were observed.

### 2.9. Differential Scanning Calorimetry

The denaturation temperature of the dECM samples was examined using a differential scanning calorimeter (DSC 4000, Perkin Elmer, Waltham, MA, USA). Powdered dECM samples, each weighing 5 mg (*n* = 3), were analyzed. The analysis involved a temperature scan ranging from 30°C to 300°C, with a heating rate of 10°C/min under a nitrogen atmosphere [31].

### 2.10. Thermal Gravimetric Analysis

The thermal degradation of the dECM and raw viscera was investigated through a thermogravimetric analyzer (DTG-60H, Shimadzu, Kyoto, Japan). The lyophilized raw and decellularized samples weighing approximately 10.0 mg were placed in an alumina pan. Temperature scans of 30°C to 700°C at a rate of 10°C/min, with an air influx of 20 mL/min [34] were conducted for all the samples.

### 2.11. Amino Acid Analysis

The amino acid composition was determined following the method of Bilgin et al. [35] with slight modifications. A 125 mg dECM sample was hydrolyzed with 5 mL of 6 N HCl at 110°C for 24 hours. After hydrolysis, the samples were cooled to room temperature and then centrifuged at 4000 rpm for 5 minutes to remove particulate matter. The hydrolysates were filtered for further purification using a 0.22 *μ*m pore size syringe filter. The filtered hydrolysates were diluted in a 1 : 10 ratio with filtered deionized water and placed into sample vials. The amino acid content was analyzed using high-performance liquid chromatography (Prominence-I Plus System, Shimadzu, Kyoto, Japan), equipped with a C18 column and a fluorescence detector. Post-run data processing was performed using LabSolutions LC software.

### 2.12. Residual Detergents Determination

The residual detergent in the dECM samples was determined using a Victor Nivo Alpha F Multimode plate reader (PerkinElmer, USA). For residual TX100 determination, the quantification method followed from Pavlović et al. [36] with slight modifications to the standards. Initially, 1 g of lyophilized samples were soaked in 10 mL of 50% methanol and then subjected to centrifugation. The resulting supernatant was decanted and subsequently analyzed at a wavelength of 225 nm. Conversely, residual SDS was analyzed using a methylene blue assay adapted from Alizadeh et al. [37]. Lyophilized samples were soaked in 5 mL of water for 24 hours and diluted with 10 mL of 75% ethanol. The solution was then filtered, mixed with a methylene blue solution and chloroform, and analyzed for residual SDS at a wavelength of 650 nm.

### 2.13. Lipid Extraction

The total lipid content of the raw and dECM samples was determined using a modified Folch method [38]. A 0.5 g tissue sample was homogenized with 10 mL of a chloroform/methanol mixture (2 : 1 ratio) and agitated for 15 minutes using an orbital shaker (Jintan Medical Instrument Factory, China) at RT. The mixture was then vortex-mixed for 2 minutes and centrifuged at 2000 rpm to separate the two-phase system. After extraction, 2 mL of water was added per 10 mL of the solvent mixture to wash the solvent, followed by vortexing to ensure complete washing. The tubes were centrifuged again at 2000 rpm until clear phase separation was observed. After centrifugation for 5 minutes, the organic phases were carefully collected, and the solvent was removed using a rotary evaporator. Finally, the obtained lipids were weighed to determine their content for each sample.

### 2.14. Statistical Analysis

The quantitative data were expressed as the mean value ± standard deviation (SD) and were subjected to a one-way analysis of variance (ANOVA). A post hoc Tukey HSD test was then employed to determine significant differences between groups, with a *p* value <0.05.

## 3. Results and Discussion

### 3.1. Histological Staining

**Figure 1:**
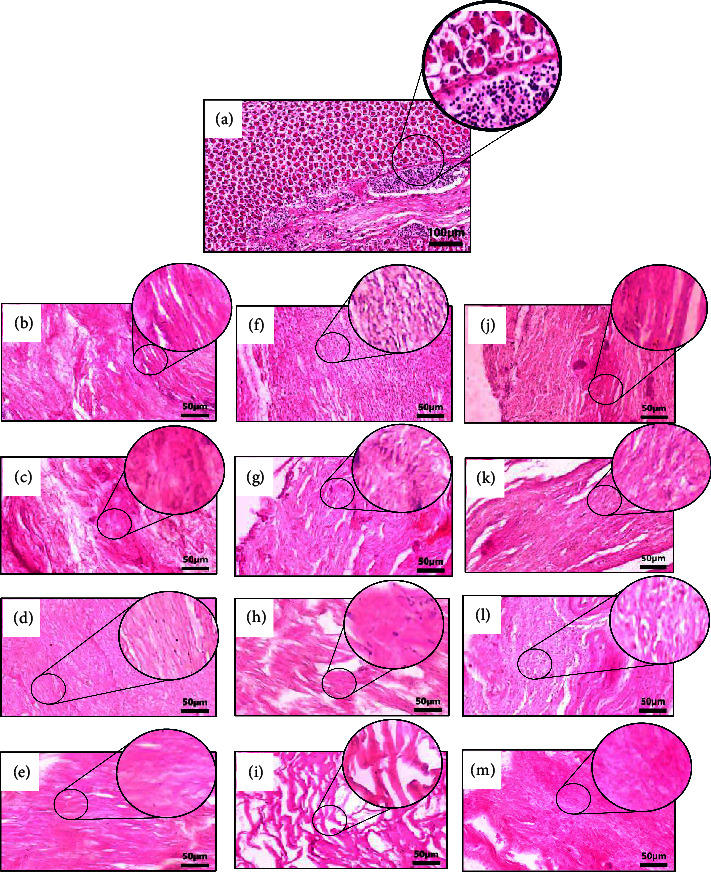
Photo microimages from the nuclear staining of the (a) raw viscera tissue and tissues decellularized with (b–e) sonication (b) 0.1% TX100, (c) 0.1% SDS, (d) 0.3% TX100, (e) 0.3% SDS; (f–i) orbital shaking (f) 0.1% TX100, (g) 0.1% SDS, (h) 0.3% TX100, (i) 0.3% SDS; and (j–m) agitation at 4 °C (j) 0.1% TX100, (k) 0.1% SDS, (l) 0.3% TX100, (m) 0.3% SDS (scale bars; (a) 100 *µ*m and (b–m) 50 *µ*m).

The H&E staining images in Figure 1 show a clear difference in cell removal between raw viscera tissue (Figure 1(a)) and decellularized samples (Figures 1(b)–1(m)). The raw tissue sample exhibits the presence of the basophilic component, which appears substantial following the deep purple staining within the tissue matrix. In contrast, the decellularized extracellular matrix (dECM) samples exhibit a significant decrease in purple staining, with some samples displaying minimal to negligible nuclear residue. Within the dECM samples, it is apparent that using 0.3% TX100 (Figures 1(d), 1(h), and 1(l)) and 0.3% SDS (Figures 1(e), 1(i), and 1(m)) leads to a notable reduction in cellular remnants. The most pronounced reduction occurs when 0.3% SDS is combined with sonication and agitation at 4°C. These findings imply that a detergent concentration of 0.3% is crucial for efficient nuclear elimination in intricate tissues like viscera. However, higher concentrations often compromise the structural integrity of the ECM [39], as noticeable by the uneven pink staining patterns, particularly in samples that undergo 24 hours of orbital shaking. Conversely, samples treated with 0.1% TX100 (Figures 1(a), 1(f), and 1(j)) and 0.1% SDS (Figures 1(c), 1(g), and 1(k)) show minimal changes in ECM structure. The effectiveness of detergent penetration in this intricate network of tissue is largely influenced by the method of decellularization used [23]. Specifically, gentle agitation at 4°C results in minimal modification of the pink staining pattern, while decellularization through sonication and orbital shaking causes more significant tissue disruption.

### 3.2. Scanning Electron Microscopy

**Figure 2:**
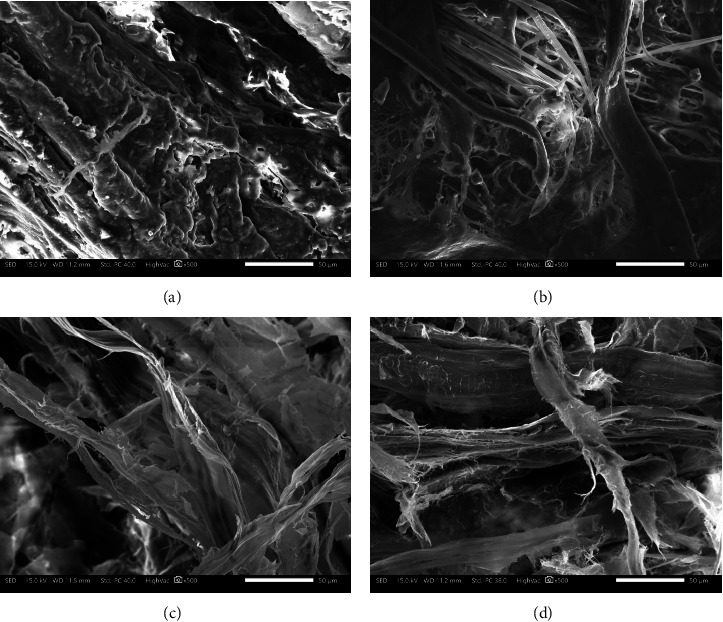
Scanning electron microscopy (SEM) images of (a) raw viscera, (b) dECM produced via agitation at 4°C at 0.3% TX100, (c) dECM produced via sonication at 0.3% TX100, and (d) dECM produced via orbital shaking at 0.3% SDS.

The microimages of raw viscera against the representative dECM samples are shown in Figure 2. The raw viscera (Figure 2(a)) exhibited a densely smooth wavy pattern, likely due to the high percentage of free fatty acids and other noncollagenous components in the raw tissue [40]. Representative dECM samples displayed morphological changes at similar magnification, appearing as dry, fiber-like tissue strands. An in-depth analysis revealed that dECM produced via agitation at 4°C in 0.3% TX100 (Figure 2(b)) displayed small interfibrillar spaces and a cluttered fiber mesh, indicating minimal alteration of the dECM structure. A thinner sheet-like structure with a cluttered fiber mesh arrangement is visible in Figure 2(c), while denser and more disorganized fiber structures are evident in Figure 2(d). The tissue swelling and structural changes observed in Figures 2(c) and 2(d) resulted from the combined effects of mechanical agitation, ultrasonication, and increasing detergent concentrations [18]. The use of 0.3% TX100 at 4°C appeared to inflict less disruption on the viscera tissue's ECM than SDS, likely due to the detergent's nonionic, uncharged hydrophilic headgroup, which allows better preservation of the ECM structure [39].

### 3.3. dsDNA and Protein Quantification

**Figure 3:**
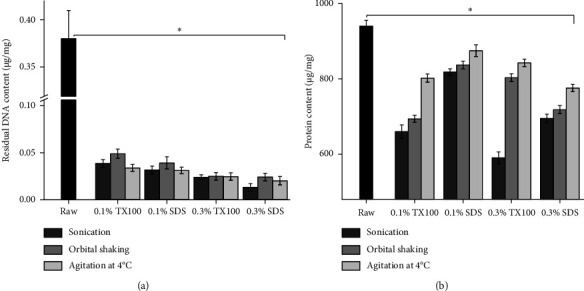
Analysis of the (a) DNA content and (b) protein content of the dECM. The horizontal line indicates a significant difference (^∗^*p* < 0.05) compared with the raw.

As illustrated in Figure 3(a), all decellularization methods significantly reduced DNA content in all dECM samples compared to raw tissue (*p* < 0.05), with all methods revealing DNA content below the acceptable residual threshold of 0.05 *µ*g/mg [23]. Sonication with 0.3% SDS for 24 hours resulted in the highest DNA removal efficiency of 96.5 ± 1.1%, suggesting that sonication disrupts cell membranes more effectively, making them more permeable to detergent action [25]. Similarly, agitation at 4°C with 0.3% SDS demonstrated a high DNA removal efficiency of 94.6 ± 1.1%, potentially due to the low temperature agitation inducing a quasi-frozen state in tissues, leading to cellular lysis and enhanced detergent permeation [41]. Comparing different physical treatments revealed no significant differences (*p* < 0.05), suggesting that sonication, agitation, and orbital shaking primarily aid in detaching cells from the matrix. Still, additional treatment, such as the integration of detergents, is needed for effective cellular removal. Conversely, a significant difference was observed between samples treated with 0.1% and 0.3% concentrations (*p* < 0.05), with increased DNA removal observed at higher concentrations. Thus, it is evident that the heightened disruption of cellular membranes induced by sonication, shaking, and agitation may also result from enhanced penetration of the detergent in the sample, resulting in elevated levels of DNA removal [39].

Decellularization processes aim to preserve proteins, which are pivotal for cell proliferation, differentiation, migration, and function [31, 37]. The rich protein content of tilapia viscera as a source for ECM [42, 43] was the primary motivation for this study. The results in Figure 3(b) show protein content variations, all of which were significantly different from the protein content observed in the raw tissue (*p* < 0.05). The comparison of physical treatments varied based on the decellularizing agent used and its concentration. For samples treated with the sonication method, an increase in the decellularizing agent concentration from 0.1% to 0.3% resulted in reduced protein content. In contrast, samples treated with orbital shaking and agitation at 4°C exhibited different trends: higher TX100 concentrations correlated with increased protein content, whereas higher SDS concentrations led to decreased protein content. The difference in protein retention between methods could be associated with the combined effect of the physical method and detergent action in disrupting proteins. The similar decreasing trends observed with orbital shaking and agitation at 4°C could be attributed to the similarity in their tissue disruption mechanisms, which involve oscillating or rotating in an orbital pattern. The effect of sonication may be due to the evenly distributed ultrasonic waves over a given period, increasing the potential for mechanical damage to the matrix. Among the dECM samples, the least reduction in protein content was observed after agitation at 4°C in 0.1% SDS, with an estimated amount of 875 ± 15 *µ*g/mg of protein remaining, followed by agitation at 4°C in 0.3% TX100, with around 842 ± 9 *µ*g/mg of protein remaining after the process. Ren et al. [44] suggested that utilizing TX100 as a posttreatment in the decellularization process is more adept at preserving ECM protein components, indicating that agitation at low temperatures in TX100 induces less disruption to the ECM structure.

### 3.4. Attenuated Total Reflectance—Fourier Transform Infrared (ATR–FTIR) Spectroscopy

**Figure 4:**
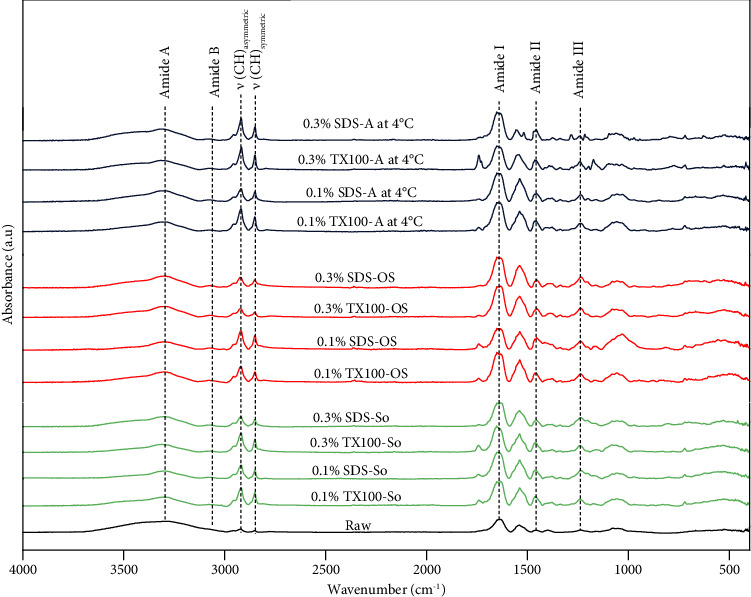
ATR-FTIR spectra of the different decellularization methods. Green spectra refer to sonication, red spectra refer to orbital shaking, and blue spectra refer to agitation at 4°C. Sonication (So), orbital shaking (OS), agitation at 4°C (A at °C) to indicate abbreviations in the spectra.

FTIR analysis revealed different characteristics between the raw tilapia viscera and decellularized extracellular matrix (dECM) samples (Figure 4). The raw tissue spectrum displayed broader, lower intensity peaks compared to the relatively developed spectra patterns, especially for the characteristic peaks of collagen such as Amide A, Amide B, Amide I, Amide II, and Amide III. The Amide A band, characterized by N-H stretching vibrations, was observed between 3310 and 3280 cm^−1^ in this study. This peak falls within the typical range (3000–3500 cm^−1^) associated with intermolecular hydrogen bonding [45–47]. The Amide B, associated with N-H stretching and its asymmetrical and symmetrical stretch CH_2_ vibrations were observed at around 3073 cm^−1^, 2950 cm^−1^, and 2845 cm^−1^, respectively [48, 49]. Spectra peaks between 1650 and 1654 cm^−1^ were also observed, indicating the C=O stretching vibrations of carbonyl groups of the Amide I region [48, 50]. Vibrational bands were observed at 1409 cm^−1^ and 1270 cm^−1^, corresponding to N-H bending vibrations associated with CN stretching relative to CH_2_ bending (amide II) and C-O stretching (amide III), respectively [49]. The presence of these various amides; amide A or *ν* (NH), amide B or *ν* (NH), amide I or *ν* (C=O), amide II or *δ* (NH), and amide III or *δ* (CH_2_) aligns with findings reported from the swim bladders of other marine species such as the Atlantic cod [47] and yellowfin tuna [51].

Guzzi Plepis et al. [52] suggest that the confirmation of collagen's triple helical structure can be evaluated by calculating the absorbance ratio between the amide II and amide III bands. As shown in Table 2, all decellularization methods yielded ratios close to 1.0. This suggests minimal disruption of the collagen triple helix within the extracellular matrix (ECM) during the extraction process. Doyle et al. [53] reported that hydrogen bonding with the NH group of amide A in a peptide causes a shift to lower frequencies, around 3300 cm^−1^. Consistent with this, the amide A band in all decellularized samples shifted toward lower frequencies. This implies a greater involvement of NH groups in hydrogen bonding throughout the process, which likely plays a crucial role in maintaining the triple helical structure of collagen within the ECM.

**Figure 5:**
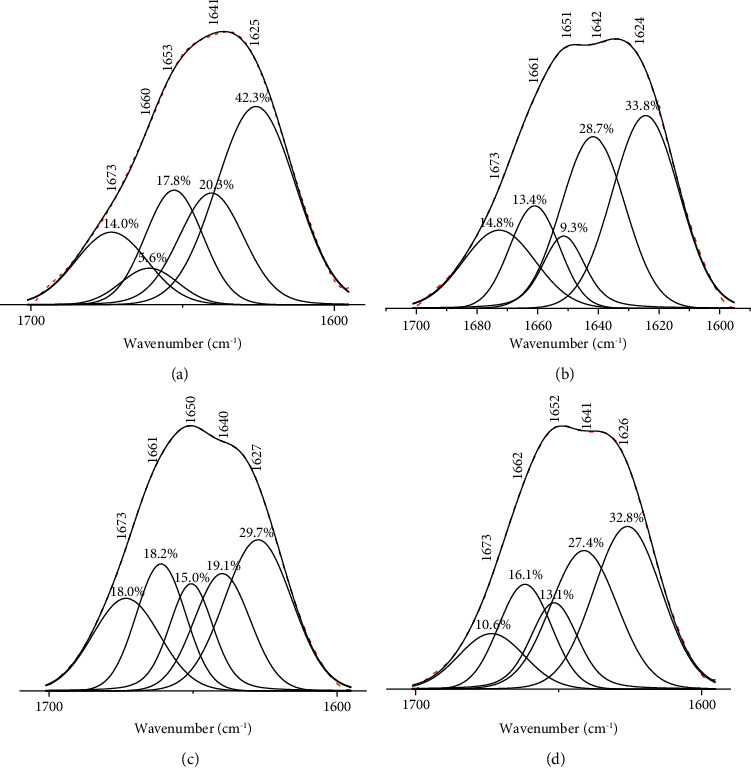
The deconvolved spectra of the amide I absorbance bands of (a) raw viscera, (b) dECM via agitation at 4°C at 0.3% TX100, (c) dECM via sonication at 0.3% TX100, and (d) dECM produced via orbital shaking at 0.3% SDS.

The amide I band, typically located between 1700 cm^−1^ and 1600 cm^−1^, primarily reflects vibrational modes of coupled carbonyl stretching [56, 57]. This band serves as a valuable marker for analyzing the secondary structures of collagen. Using a combination of Gaussian and Lorentzian fitting methods (Figure 5, Table 3), the analysis further revealed the estimated distribution of the collagen secondary structures such as *α*-helices, *β*-sheets, *β*-turns, and random coils of both raw and dECM samples. The raw tissue exhibited a lower proportion of *α*-helix (5.6%) but had relatively higher amounts of *β*-sheets, *β*-turns, and random coils than the dECM samples. The significant presence of *β*-sheets in the samples indicates fewer intermolecular hydrogen bonds, resulting in a less stable secondary structure [58]. This instability may be due to or influenced by the various noncollagenous compounds present alongside collagen in the raw tissue.

In contrast, a higher *α*-helix content is typically associated with increased strength and stability within collagen structures, which are crucial for maintaining the integrity and mechanical properties of the extracellular matrix [54, 57]. As shown in Table 3, the change in *α*-helix content was influenced by the type and concentration of detergent used during decellularization. Treatment with TX100 generally resulted in unstable *α*-helices, showing a rapid increase, especially in samples treated with sonication and orbital shaking. In contrast, SDS treatment generally resulted in a stable increase, with no substantial fluctuation in *α*-helix values. This implies that increasing the TX100 concentration may disrupt the matrix. However, it could also mean that this detergent can be more effective in removing noncollagenous components of the raw tissue if an appropriate concentration is used in the process.

### 3.5. SDS-PAGE Pattern

**Figure 6:**
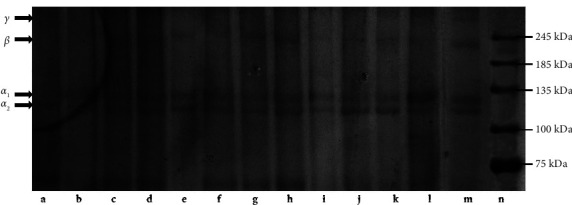
SDS-PAGE electrophoretic pattern of the (a) raw viscera tissue and tissues decellularized with (b–e) sonication (b) 0.1% TX100, (c) 0.1% SDS, (d) 0.3% TX100, (e) 0.3% SDS; (f–i) orbital shaking (f) 0.1% TX100, (g) 0.1% SDS, (h) 0.3% TX100, (i) 0.3% SDS; and (j–m) agitation at 4°C (j) 0.1% TX100, (k) 0.1% SDS, (l) 0.3% TX100, (m) 0.3% SDS, and (n) marker.

The assessment of the protein eluted in all the dECM samples was examined by gel electrophoresis as shown in Figure 6. As depicted in the result, all samples including the raw tissue exhibited similar electrophoretic patterns, revealing four specific bands. The following bands were identified as the monomers of (*α*1 and *α*2) chains which occurred in this study at around 120–125 kDa and 130–135 kDa, respectively. Consequently, when the two monomers (*α*1 and *α*2) chain ratio would be 2 : 1, which our results displayed similar outcomes, could suggest that the collagen presence of the viscera is a type I collagen [51] which supports the Amide I deconvolution results. However, of the sample eluted, the clear separation of the *α*1 and *α*2 monomers was visible when agitation at 4°C using 0.3% TX100 was employed as the decellularization method. This indicates that dECM from 0.3% concentration has undergone partial cleavage at the telopeptide where most of the intra- and intermolecular crosslinks are found in collagen [59]. Additionally, two other bands of higher molecular weight were observed, identified as the dimer (*β* chain) (228–233 kDa) and trimer (*γ* chain) (257–263 kDa), which were also previously identified as collagen protein bands of tilapia skin [60]. These results were also identical to the protein patterns found in the swim bladders of yellowfin tuna [51], seabass [61], carp fish scale [62] bigeye snapper [63] grass carp, and tilapia skin collagen [60].

### 3.6. Differential Scanning Calorimetry (DSC)

**Figure 7:**
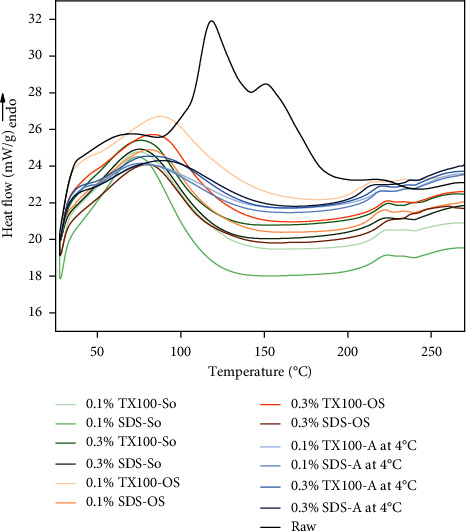
Differential scanning calorimetry curves of raw tissue and the dECM samples.

As illustrated in Figure 7, the raw viscera exhibited four denaturation temperatures (TD), comprising three major peak ranges (37–85°C), (107–142°C), (207–239°C), and one shoulder peak range (140–150°C), a characteristic attributed to the presence of fat in the viscera. Other peaks were due to the evaporation of water-soluble substances, degradation of the lipid-soluble fraction, and denaturation of ECM molecules [31, 64]. This curve pattern was similar to the thermal properties of porcine adipose tissue [64], suggesting a substantial amount of lipid-soluble substance in raw tissue. However, all decellularized samples revealed only two TD occurring between (50–115°C) and (205–245°C), respectively, which can be associated with the reduction of lipid-soluble components, leaving behind only the denatured ECM molecules and water-soluble substances [65].

The first major endothermic peak observed in all decellularized samples exhibited higher temperature when compared to the denaturation of collagen around (35–40°C) [65]. In the agitation at 4°C method, the first peaks were shifted further to the right (80–100°C), with a much broader peak relative to the raw tissue and decellularized sample. Whereas orbital shaking has endothermic peaks from (80–95°C), slightly lower end set temperature from agitation at 4°C. The sonication method shows a slight increase in TD peak, around (70–75°C) and a sharp peak accordingly. The shifting to a higher temperature and broadening of the peak of the TD were the effects of the crosslinking of the molecules where its network is surrounded by H-bonded water molecules that increase the thermal stability of the ECM [65, 66]. In addition, TX100-treated samples have a higher peak of thermal denaturation than SDS-treated samples.

The second endothermic peak, detected within the temperature range of (220–235°C), exhibited a similar trend to that observed in the raw sample, which is associated with the breaking of the hydrogen bond left behind and the result of the release of structural moisture within the ECM for both raw and treated samples [31, 65].

### 3.7. Thermogravimetric Analysis (TGA)

**Figure 8:**
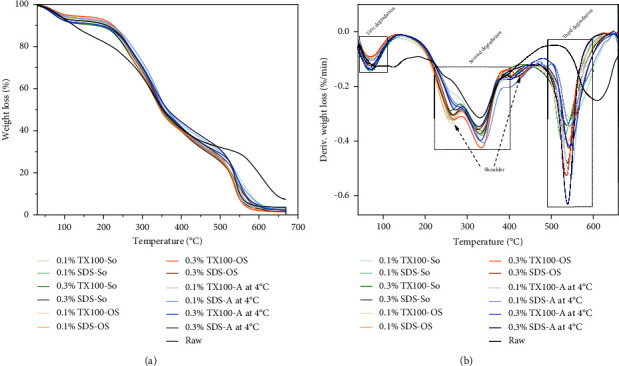
Thermogravimetric curves (a) weight loss and (b) differential TG (dTGA) of raw and dECM samples.

Figure 8(a) shows the weight loss curve of the raw and all decellularized samples which exhibited a small difference in thermal degradation pattern and had an end set temperature lesser than that of the raw tissue. The degradation peaks of the dECM were emphasized in the derivative thermogravimetric (dTGA) curves, as shown in Figure 8(b). All decellularized samples exhibited a notably distinctive three-step weight loss curve, where the second step happened to have a shoulder at around (152–287°C) before the second peak.

The primary step is associated with the evaporation of strongly H-bonded water molecules that persisted in the dECM samples [60]. The average percentage mass loss observed in the decellularized sample suggests that the water content in each dECM sample remained nearly identical, even after undergoing a different decellularization process. Further increased temperature transitioned to a two-step degradation curve, which happened to be the step with the greatest mass loss area. The onset temperature at (152°C) in the shoulder is associated with changes in the conformation of collagen molecules, transitioning from a triple helix structure to a random coil [67, 68]. The degradation peaks in this decomposition step occurring at (260°C) and (330°C), respectively, attributed to the rapid breakdown of the protein constituting the ECM of viscera, primarily consisting of collagen. Notably, samples exposed to 0.1% TX100 sonication deviate from this thermal profile, displaying spectral characteristics like raw tissue and lacking the transitional shoulder. This observation suggests that the tertiary structure of the proteins within the sample remained unaltered before thermal denaturation.

The third step occurs under the temperature range of (470–615°C), where a small shoulder also appears at (410–420°C). This shoulder corresponds to the remaining lipid that persists even after decellularization, which also correlates with the data reported by Chun et al. [69]. The highest peak in this step is associated with carbon residues that remained after heating all the volatile organic components [34, 60, 65]. Regardless of the decellularization method used, the consistent thermal decomposition patterns in all decellularized viscera samples suggest a comparable degradation rate of the ECM compositions during the process.

### 3.8. Amino Acid Profile

Table 4 displays the amino acid composition of dECM obtained from different decellularization methods. All dECM samples exhibited a significant presence of glycine, accounting for approximately 30% of the total amino acid content, closely aligning with the expected content in collagen. Moreover, high percentages of alanine, proline, asparagine, and glutamine were observed, consistent with the typical amino acids found in collagen structures [46, 60, 65]. These results resemble the amino acid profile of type I collagen found in various fish tissues such as carp fins, scales, skins, bones, and swim bladders [37], as well as other fish species like amur sturgeon [65, 70], and bigeye tuna [45]. Among the methods, agitation at 4°C with 0.3% TX100 (∼36.3%) and 0.3% SDS (∼36.7%) displayed the highest glycine content compared to orbital shaking and sonication methods. However, all of these glycine content values were much higher than those found in the raw viscera, indicating successful removal of lipids, which exposes most of the amino acid residue that makes up the protein. According to Qian et al. [71], abundant glycine could be associated with higher antioxidant activity; hence, agitation at low temperatures will be preferable in biomedical applications. The resemblances of FTIR and gel electrophoresis results on its electrophoretic movement and amino acid composition indicated a strong preservation of type I collagen's chemical structure across the tissues [72].

### 3.9. Residual Detergent Determination Test

**Figure 9:**
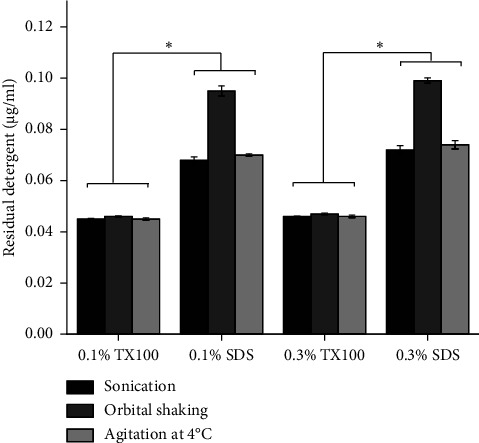
Residual detergent concentration for Triton X-100 and SDS in the three different physical decellularization methods. The horizontal line indicates a significant difference (^∗^*p* < 0.05).

The method of decellularization and the ease of removing detergent from the tissue through washing are crucial factors in the decellularization process. Inadequate removal of detergents can negatively impact subsequent scaffold recellularization [23, 73].  Figure 9 shows that as the concentrations of the detergents increased, the residual detergent in the decellularized samples also increased. Significantly higher residual detergent concentrations were observed in dECM treated with SDS compared to those treated with TX100 (*p* < 0.05). These findings align with previous studies highlighting the challenge of removing SDS compared to other detergents like TX100 [31, 37]. This high concentration of SDS detergent residue in the dECM samples can be attributed to the adhesive nature of this anionic detergent, which intercalates into various biomolecules such as proteins [23, 74]. It has been demonstrated that the washing method employed in this study was highly effective in removing TX100.

### 3.10. Residual Lipid Test

**Figure 10:**
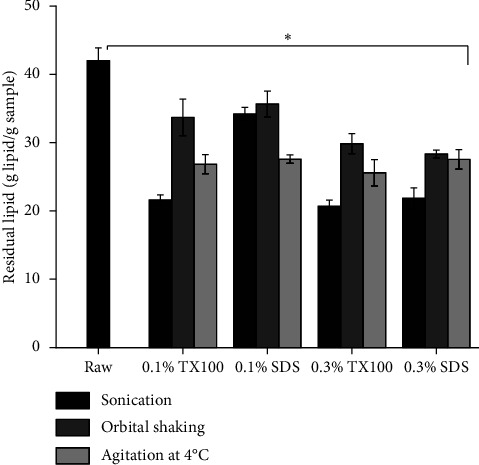
Residual lipid concentration of the raw and the dECM samples. The horizontal line indicates a significant difference (^∗^*p* < 0.05).

Decellularization protocols could be tailored to consider factors such as tissue density, cellularity, and lipid content. This is particularly crucial for tissues with high-fat content, including adipose tissue, bones, and viscera [75]. In Figure 10, the graph displays residual lipid content in decellularized extracellular matrix (dECM) samples compared to raw tissue. The results indicate a significant reduction in lipid content (*p* < 0.05) in dECM samples. Among all the protocols employed, sonication with 0.3% TX100 treatment was observed to the have most significant (*p* < 0.05) reduction of residual lipid content of 20.68% ± 0.91 (dry basis), compared to 41.99% ± 1.85 (dry basis) in the raw tissue. This substantial reduction can be attributed to the effective disruption caused by ultrasonic waves during the sonication process, which creates pseudopores in the tissue membrane. Furthermore, the effectiveness of TX100 compared to SDS can be linked to its specific target mechanisms. TX100 primarily disrupts lipid-lipid interactions within the lipid membrane [76]. When combined with the tissue destruction induced by sonication, this results in enhanced lipid removal efficiency. Although sonication proved to be highly efficient, other methods such as orbital shaking and agitation at low temperatures also showed significant reductions (*p* < 0.05) in crude lipid content. This could be due to the continuous stirring for 24 hours and the effects of washing from treatment to posttreatment and incorporating lyophilization in the process [39].

## 4. Conclusion

Viscera waste possesses functional properties that have proven valuable in nutraceutical and biomedical research. However, there is currently a lack of studies utilizing viscera waste as a tissue-derived extracellular matrix. Although the production of decellularized tissue is gaining popularity as an alternative, challenges remain in selecting an optimal decellularization method. To address this gap, the present study utilizes tilapia viscera as a raw material to investigate the assessment of three physical methods: sonication, orbital shaking at room temperature, and agitation at 4°C, combined with detergents (SDS and TX100) at varying concentrations (0.1% and 0.3%). Our results demonstrated that all decellularization methods successfully removed most of the cellular material while minimizing disruption of the ECM structure. However, different decellularization methods resulted varied outcomes in terms of ECM structure preservation, cellular removal, and ECM composition. Agitation at 4°C in TX100 proved efficient in preserving the ECM structure and matrix components. Meanwhile, sonication in SDS treatment shows the highest removal of cellular components. These specific combinations exhibit promising potential for achieving effective decellularization of tilapia viscera tissue. However, the most significant findings emerged from protocols employing sonication with 0.3% SDS and agitation with 0.3% TX100, meeting the criteria for effective removal of cellular components and preservation of the ECM structure and its components, respectively. Our findings strongly imply that decellularized tilapia viscera present a promising prospect as a biological scaffold for tissue reconstruction applications. Further studies are necessary to comprehensively assess the dECM developed from tilapia viscera using different decellularization methods. Additional tests will be essential to validate these results, including evaluation of mechanical properties, determination and quantification of other ECM compositions, cytotoxicity assessments, biocompatibility studies, and other characterizations.

## Figures and Tables

**Table 1:** Overview of the decellularization conditions.

Method	Agent	Concentration	Contact time (hrs)	Temperature	Washing
Sonication	SDS, TX100	0.1%, 0.3%	10	RT	15 min ×3 & 24 hrs
Orbital shaking			24	RT	
Agitation			24	4 °C	

**Table 2:** The absorption ratio (amide III: amide II) obtained from FTIR spectra.

Decellularization protocols		1242.16 cm^−1^/1462.04 cm^−1^ bond ratio
Sonication	0.1% TX100	0.95
	0.3% TX100	0.98
	0.1% SDS	0.99
	0.3% SDS	1.02

Orbital shaking	0.1% TX100	0.95
	0.3% TX100	1.02
	0.1% SDS	0.95
	0.3% SDS	1.03

Agitation at 4°C	0.1% TX100	0.83
	0.3% TX100	0.98
	0.1% SDS	0.97
	0.3% SDS	0.95

**Table 3:** Percent relative absorbance of the amide I band components of raw and decellularized extracellular matrix sample (dECM).

Amide I position (cm^−1^)^a^	Assignment	Raw	Secondary structure (%)											
			Sonication				Orbital shaking				Agitation at 4°C			
			0.1% SDS	0.3% SDS	0.1% TX100	0.3% TX100	0.1% SDS	0.3% SDS	0.1% TX100	0.3% TX100	0.1% SDS	0.3% SDS	0.1% TX100	0.3% TX100
1674–1695	Beta sheet (Antiparallel)	42.3	26.5	37.0	23.5	29.7	25.5	32.8	23.3	32.3	34.9	33.0	30.7	33.8
1662–1686	Beta turns	14.0	14.2	9.4	15.9	18.0	13.3	10.6	12.3	10.3	16.7	13.9	12.8	14.8
1648–1660	Alpha helix	5.6	15.9	15.4	14.6	18.2	16.5	16.1	13.3	15.8	13.9	13.1	16.0	13.4
1642–1657	Random coil	17.8	18.5	20.4	7.7	15.0	14.9	13.1	6.57	16.8	9.65	7.31	18.9	9.3
1623–1641	Beta sheet (parallel)	20.3	24.8	17.7	38.2	19.1	25.5	27.4	44.6	24.7	24.8	32.6	21.5	28.7

^a^Information source from refs [32, 54, 55].

**Table 4:** Amino acid composition of raw viscera tissue and decellularized samples.

Amino acids	Raw viscera	Sonication				Orbital shaking				Agitation at 4°C			
		0.1% SDS	0.3% SDS	0.1% TX100	0.3% TX100	0.1% SDS	0.3% SDS	0.1% TX100	0.3% TX100	0.1% SDS	0.3% SDS	0.1% TX100	0.3% TX100
Gly	21.4	30.6	29.3	29.9	32.0	30.1	34.6	35.7	35.5	33.3	36.7	34.8	36.3
Val	2.7	1.8	2.0	2.0	2.0	1.9	1.5	1.7	1.4	1.8	1.6	1.7	1.7
Glu	4.9	6.6	6.9	6.8	6.5	6.9	6.5	6.0	6.9	6.7	6.7	6.5	6.3
Asp	9.3	8.0	7.4	8.2	7.4	7.3	6.9	7.3	6.8	7.3	6.4	6.8	6.6
Ala	10.2	13.6	12.7	12.4	12.8	13.1	12.8	12.5	12.8	12.5	12.6	12.2	12.1
Ser	6.7	5.3	5.1	5.0	5.3	6.3	6.1	5.9	6.0	6.1	5.9	6.0	5.8
Thr	4.0	2.9	3.2	3.1	3.2	3.3	3.8	3.4	2.8	3.0	3.1	3.2	3.1
Arg	5.2	6.6	6.1	6.0	6.2	6.1	6.0	5.9	5.4	6.0	5.5	5.3	5.2
Lys	3.5	1.9	2.5	2.2	2.0	2.2	2.0	1.9	2.0	2.2	1.8	2.0	1.8
Leu	5.6	3.7	3.9	3.9	3.8	3.8	3.2	3.6	3.0	3.7	3.0	3.3	3.2
Met	2.0	1.6	1.7	1.5	1.3	1.3	1.0	1.2	1.2	1.4	1.0	1.1	1.0
Phe	2.7	2.2	2.2	2.4	2.2	2.1	1.9	2.1	1.9	2.1	1.9	2.0	1.9
Ile	2.2	1.5	1.7	1.5	1.4	1.3	1.1	1.2	1.0	1.3	0.9	1.1	1.0
Tyr	1.7	2.7	3.5	2.4	2.8	0.3	1.8	3.3	0.9	3.7	3.0	4.0	4.9
His	1.2	0.7	0.8	0.8	0.8	0.9	0.9	1.0	0.6	0.8	0.8	0.8	0.7
Cys	0.9	1.1	1.0	1.0	1.0	1.0	0.9	0.8	1.1	1.0	0.8	0.9	2.0
Pro	11.7	10.4	10.1	10.7	9.6	9.6	9.1	9.2	8.8	9.7	8.4	8.6	8.3

Values are computed in percent concentrations.

## Data Availability

The data required to reproduce the above findings cannot be shared at this time as the data also forms part of an ongoing study.
